# The Effect of Aging on Relationships between Lean Body Mass and VO_2_max in Rowers

**DOI:** 10.1371/journal.pone.0160275

**Published:** 2016-08-01

**Authors:** Chul-Ho Kim, Courtney M. Wheatley, Mehrdad Behnia, Bruce D. Johnson

**Affiliations:** 1 Human Integrative and Environmental Physiology Laboratory, Mayo Clinic, Rochester, Minnesota, United States of America; 2 Cardiopulmonary Laboratory, Doctors Hospital, Augusta, Georgia, United States of America; University of Rome Foro Italico, ITALY

## Abstract

Aging is associated with a fall in maximal aerobic capacity as well as with a decline in lean body mass. The purpose of the study was to investigate the influence of aging on the relationship between aerobic capacity and lean body mass in subjects that chronically train both their upper and lower bodies. Eleven older rowers (58±5 yrs) and 11 younger rowers (27±4 yrs) participated in the study. Prior to the VO_2_max testing, subjects underwent a dual energy X-ray absorptiometry scan to estimate total lean body mass. Subsequently, VO_2_max was quantified during a maximal exercise test on a rowing ergometer as well as a semi-recumbent cycle ergometer. The test protocol included a pre-exercise stage followed by incremental exercise until VO_2_max was reached. The order of exercise modes was randomized and there was a wash-out period between the two tests. Oxygen uptake was obtained via a breath-by-breath metabolic cart (Vmax^™^ Encore, San Diego, CA). Rowing VO_2_max was higher than cycling VO_2_max in both groups (p<0.05). Older subjects had less of an increase in VO_2_max from cycling to rowing (p<0.05). There was a significant relationship between muscle mass and VO_2_max for both groups (p<0.05). After correcting for muscle mass, the difference in cycling VO_2_max between groups disappeared (p>0.05), however, older subjects still demonstrated a lower rowing VO_2_max relative to younger subjects (p<0.05). Muscle mass is associated with the VO_2_max obtained, however, it appears that VO_2_max in older subjects may be less influenced by muscle mass than in younger subjects.

## Introduction

Maximal oxygen consumption (VO_2_max) is the maximum capacity to transport and utilize oxygen and is often used as a measure of an individual’s aerobic capacity. Generally, VO_2_max decreases gradually with advancing age, and the rate of decline is approximately 10% per decade after the age of 25 years, and more specifically was suggested to be 15% between the ages of 50 and 75 [1, 2, 3]. In addition, a previous meta-analysis illustrated that age-related declines in VO_2_max were approximately 0.40, 0.39 and 0.46 ml/kg/min per year for sedentary, active and trained males respectively and 0.35, 0.44, 0.62 ml/kg/min per year for sedentary, active and trained female respectively [4, 5, 6]. Age-related decline in VO_2_max results from multiple factors: decreased maximal heart rate and stroke volume, reductions in blood volume due to pooling from less effective muscle pump action of the valves in the extremities, and stiffening of both the heart muscle fibers and thickening and stiffening of the arterial walls, reduced peripheral oxygen extraction and maximal A-V O_2_ difference [7, 8, 9, 10]. Age-related muscle loss (sarcopenia) also appears to play an important role in the decline in VO_2_max; by the age of 50 about 10% of muscle area is lost and this rate only increases in the decades that follow. In concert with sarcopenia, muscle strength declines by 15% per decade in the 60s and 70s and doubles to 30% after 80 [11]. A previous study by Fleg and Lakatta (1988) demonstrated that whole body lean mass contributed to a decline in VO_2_max with advancing age in sedentary individuals [12]. Similar results were observed in active individuals [13, 14]. However, active individuals demonstrated a slower rate of decline than sedentary individuals [3, 15]. Thus the influence of muscle mass on VO_2_max can be influenced by training and possibly type of training (e.g., upper vs lower extremity exercise) as well as other factors, such as how muscle mass is quantified (e.g., only active muscles vs total lean body or skeletal muscle mass) [13].

Rowing is a demanding sport requiring a high aerobic capacity and the ability to sprint; yet it is a common sport amongst older individuals. It is an activity requiring large muscle mass and the ability to distribute blood flow to both upper and lower extremity skeletal muscles simultaneously. Accordingly, rowers exhibit both upper and lower body muscle adaptations, and thus offer a unique population to better understand the effect of muscle mass on VO_2_max.

The purpose of study was to investigate the relationship between active muscle mass and VO_2_max, and how this relationship might be influenced by age. Two separate exercise modes–recumbent cycling (targeting leg muscles only) and rowing (targeting leg and upper body muscles)–were utilized to differentiate active muscle mass. A mode specific VO_2_max was quantified based on active muscle for a group of younger and older rowers.

## Methods

For the present study, 22 subjects were recruited from rowing teams in Augusta, Georgia (GA). Eleven older subjects (age = 58±5 yr, 6 males and 5 females) and 11 younger subjects (age = 27±4 yr, 6 males and 5 females) who had at least a one year of competitive rowing experience, a current baseline level of training that included rowing and typical synergistic activities–running, swimming, resistive training and etc. They performed for a minimum of one hr four times a week, and were planning to participate in seasonal rowing competitions–regattas. Subjects who had a body mass index (BMI) >30, a history of cardiovascular or pulmonary disease, an inability to perform repeated volitional level of exercise or females who were pregnant were excluded from the study. The study was approved by Mayo Clinic Institutional Review Board and Joseph M. Still Research Foundation, Inc. (on behalf of Doctors Hospital). Prior to participating, all subjects provided their written and informed consent. The baseline pulmonary function and body composition metrics for the subjects is provided in Table 1. The study protocol included two maximal oxygen consumption tests (VO_2_max) one on a semi-recumbent bike and a second on a rowing ergometer. These exercise modes are thought to recruit a different active muscle mass with cycling being regional (leg muscle only) whereas rowing is more of a whole body muscle mass. BothVO_2_max tests were on the same day, with a 90min rest period given between tests. Subjects were randomized and counterbalanced to a test order such that half the subjects completed the cycling test first and the other half completed the rowing test first. Whole body muscle mass and regional muscle mass were estimated via dual energy X-ray absorptiometry (DEXA) scan. Quantification of specific regional muscle masses allowed for a logical estimation of active muscle mass following exercise modes. For the cycling test the representative active muscle mass was leg muscle mass (LMM), and for the rowing test whole body lean muscle mass (LBM) was used.

**Table 1:** Lung function and body composition.

	Older subjects	Younger subjects
**Lung function and diffusing capacity**		
Total lung capacity (L)	5.9±1.4	7.0±1.2
Vital capacity (L)	3.9±1.1*	5.1±1.2*
Residual volume (L)	2.1±0.4	2.0±0.3
Expiratory reserve volume (L)	1.3±0.4*	1.8±0.4*
Inspiratory capacity (L)	2.75±0.7*	3.46±0.6*
RV/TLC (%)	36.8±1.8*	27.6±2.2*
Forced vital capacity (FVC) (%pre)	111±16	107±16
FEV 1(%pre)	102±17	103±17
FEV25-75 (%pre)	78±22	93±22
Diffusing capacity for CO (%pre)	119±21	116±17
Alveolar gas volume (L)	6.1±1.5	7.0±1.3
**Body composition**		
Total mass (kg)	74.4±16	78.6±11
Body mass index	25.4±4	24.1±3
Percent body fat (%)	29.4±13	21.1±1
LBM (kg)	50.3±13	58.9±10
Fat free mass (kg)	53.2±13	62.7±10
LM (kg)	16.3±4	19.4±3
Arms lean mass (kg)	5.9±2	6.8±2

* indicates a significant difference between older and younger rowers (p<0.05).

Respiratory exchange ratio (no exertion ~ maximal exertion: 6 ~ 20).

Dyspnea (no breathlessness at all ~ maximum: 0 ~ 10).

All experimental trials and DEXA scans were conducted in Doctors Hospital (Augusta, GA). Subjects reported to the laboratory either on the same day of testing or prior to testing for review of the study protocols and signing consent form. In addition, they completed simple questionnaires of health (PAR-Q: physical activity readiness questionnaire) and activity levels (IPAQ SF: short from of international physical activity questionnaire), and female subjects competed a urine pregnancy test. Next subjects underwent a DEXA scan for body composition and a basic spirometry including single breath diffusion capacity for carbon monoxide (Vmax^™^ Encore, San Diego, CA). This was then followed by the VO_2_max tests on a rowing ergometer (Concept 2, Inc., Morrisville, VT) and semi-recumbent cycling ergometer (Lode B. V., Groningen, Netherlands).

The VO_2_max testing protocol included a warming-up stage (3min resting and 2min warm-up with 25W) followed by incremental exercise to volitional exhaustion. The exercise intensity was increased every 2min, with the first three stages consistent for all subjects (50W, 75W and 100W respectively). To ensure all subjects had a similar time to exhaustion, power output during the subsequent stages was increased by 20W for old females, 30W for old males and young females and 40W for young males until a termination of exercise.

Subjects were asked to maintain a pedal rate of 60–80 rpm for cycling and a stroke rate of 20–30 for rowing with exhaustion classified as the inability to maintain this rate despite verbal encouragement. During cycling on a recumbent cycle ergometer, the subjects were asked not to hold the handle to minimize the involvement of upper body muscle groups. Initial mode of testing were randomized and counter-balanced. Oxygen uptake and gas exchange was measured via breath by breath metabolic cart (Vmax^™^ Encore, San Diego, CA). The VO_2_max was determined as the highest 60sec average VO_2_ observed within the last 2min of incremental exercise. Heart rate was monitored via electrocardiography (ECG) and peripheral oxygen saturation via forehead probe pulse-oximeter. Perceived exertion (RPE) was obtained every stage using the Borg Scale and dyspnea by Borg’s Breathlessness Scale. A true VO_2_max was verified to have been reached when two of the following three were observed: peak heart rate (HR)> 90% predicted max (220-age), a rating of perceived exertion (RPE) 18 or greater out of 20, or a respiratory exchange ratio (RER) greater than or equal to 1.15.

To compare baseline pulmonary function and body composition between older and younger subjects, independent t-tests were conducted. To estimate the differences in VO_2_max between groups and exercise modes, repeated measures analysis of variance was conducted and subsequently paired-sample t-tests and independent t-tests were conducted. Furthermore, the relationships between VO_2_max (l/min) and active muscle mass for both exercise modes and the relationship between age and exercise modes for VO2max (corrected by LBM or LMM) were analyzed. For cycling, absolute VO_2_max (l/min) was corrected for leg muscle mass (LMM) and illustrated with *ml/kg-LMM/min*, while for rowing it was corrected with whole body muscle mass (LBM) and illustrated with *ml/kg-LBM/min*. The significance level was set at 0.05.

## Results

Peak heart rate (HR), respiratory exchange ratio (RER), rate of perceived exertion (RPE) and dyspnea obtained during cycling and rowing were illustrated in Table 2. While older subjects demonstrated no significant difference in peak HR during cycling and rowing (p>0.05), younger subjects showed a significant difference (p<0.05). In addition, both older and younger groups demonstrated no significant difference in peak HR during cycling (p>0.05), however, older subjects reached significant lower peak HR during rowing than younger subjects (p<0.05). Both older and younger subjects demonstrated a higher RER at peak during cycling than rowing (p<0.05 and p<0.05 respectively). However, no significant differences in RPE were observed between cycling and rowing in either older (p>0.05) or younger (p>0.05) subjects. Furthermore, older subjects showed no significant difference in dyspnea score at peak exercise between exercise modes (p>0.05), however, younger subjects reported higher scores during rowing (p<0.05).

**Table 2:** The assessment of exertion at peak level.

	Older subjects	Younger subjects
	Cycling vs. Rowing	Cycling vs. Rowing
Peak heart rate (beat/min)	164±10 vs. 167±7	173±6 vs. 180±8*
Respiratory exchange ratio (RER)	1.26±0.10 vs. 1.18±0.08*	1.17±0.09 vs. 1.15±0.08*
Rating of perceived exertion (RPE)	18.5±1.1 vs. 18.6±1.1	18.3±0.8 vs. 18.7±1.3
Dyspnea	7.9±1.6 vs. 7.9±1.6	7.5±1.7 vs. 8.5±1.3*
SpO_2_ (%)	98.4±1.5 vs. 97.6±1.7	98.2±1.2 vs. 96.4±1.3*
Peak power (watts)	186.4±61.9 vs. 186.4±61.9	251.8±41.9 vs. 261.1±47.8

* indicates a significant difference between cycling and rowing (p<0.05.)

Respiratory exchange ratio (no exertion ~ maximal exertion: 6 ~ 20).

Dyspnea (no breathlessness at all ~ maximum: 0 ~ 10).

Oxygen saturation (SpO_2_)

**Fig 1:**
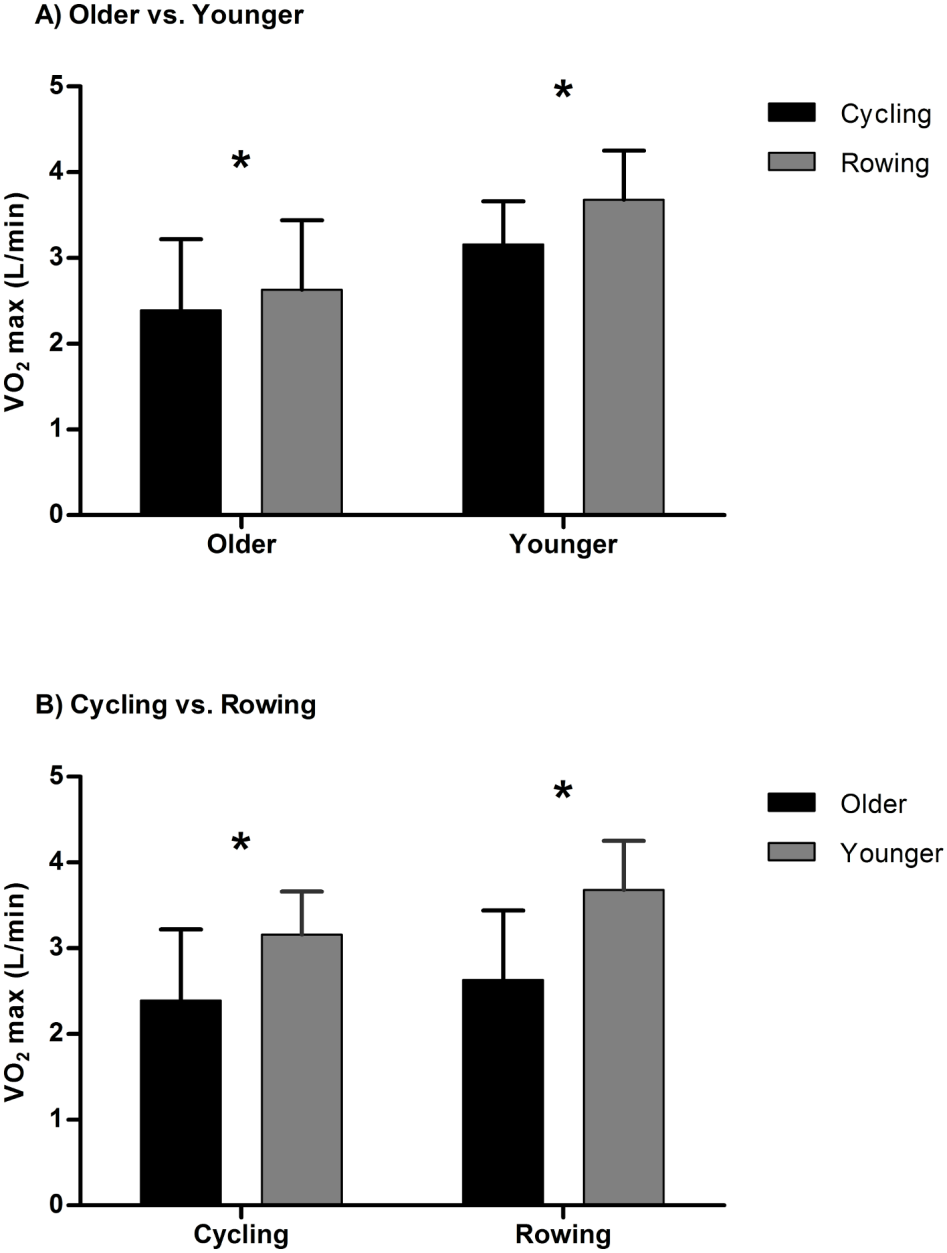
(A) The differences in VO_2_max between cycling and rowing in older and younger. (B) The differences in VO_2_max between older and younger subjects in cycle and rowing. * denotes a significant difference between exercise modes (A) and groups (B).

Both older and younger subjects had higher VO_2_max (l/min) values with rowing than cycling, however, the effect of mode of exercise, difference in VO_2_max (l/min) between cycling and rowing was significantly less in older subjects relative to the difference noted in younger subjects (p<0.05). Older subjects increased VO_2_max (l/min) by 10% from cycling to rowing (Fig 1A, p<0.05), while younger rowers improved VO_2_max (l/min) by 16.7% (Fig 1A, p<0.05). In addition, VO_2_max (l/min) of younger subjects was 29.8% higher during cycling (Fig 1B, p<0.05) and 37.6% higher during rowing (Fig 1B, p<0.05) when compared to older rowers.

**Fig 2:**
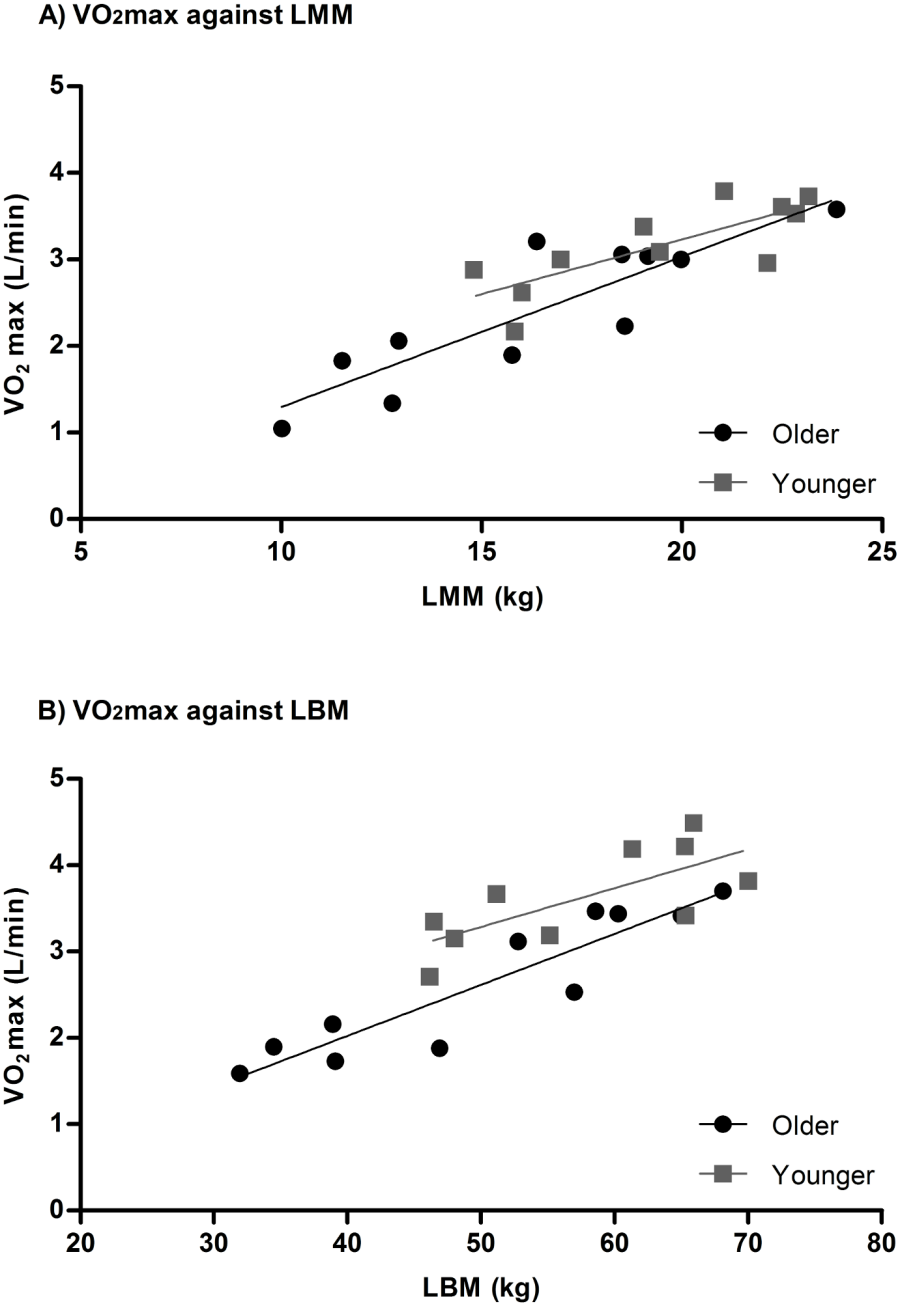
Individual VO_2_max against muscle mass. The slope of relationship between LMM and cycling VO_2_max was not significant different between older (y = 0.1737x-0.4418) and younger (y = 0.1263x+0.7057) subjects (p>0.05). The slope of relationship between LBM and rowing VO_2_max was not significant different between older (y = 0.05907x-0.3382) and younger (y = 0.04480x+1.047) subjects (p>0.05).

The relationships between cycling VO_2_max (l/min) and LMM in older (r^2^ = 0.764, p<0.05) and younger subjects (r^2^ = 0.613, p<0.05) are illustrated Fig 2A. In addition, the relationship between rowing VO_2_max and LBM in older (r^2^ = 0.851, p<0.05) and younger subjects (r^2^ = 0.531, p<0.05) are illustrated in Fig 2B.

There was a modest relationship between cycling VO_2_max (ml/kg-LMM/min) and age (r^2^ = 0.218, p<0.05) while a strong relationship between rowing VO_2_max (ml/kg-LBM/min) and age (r^2^ = 0.521, p<0.01) was observed. In addition, rowing VO_2_max (ml/kg-LBM/min) in older subjects remained lower than younger subjects (p<0.05). However, there was no longer a difference in cycling VO_2_max (ml/kg-LMM/min) between groups (p>0.05).

**Fig 3:**
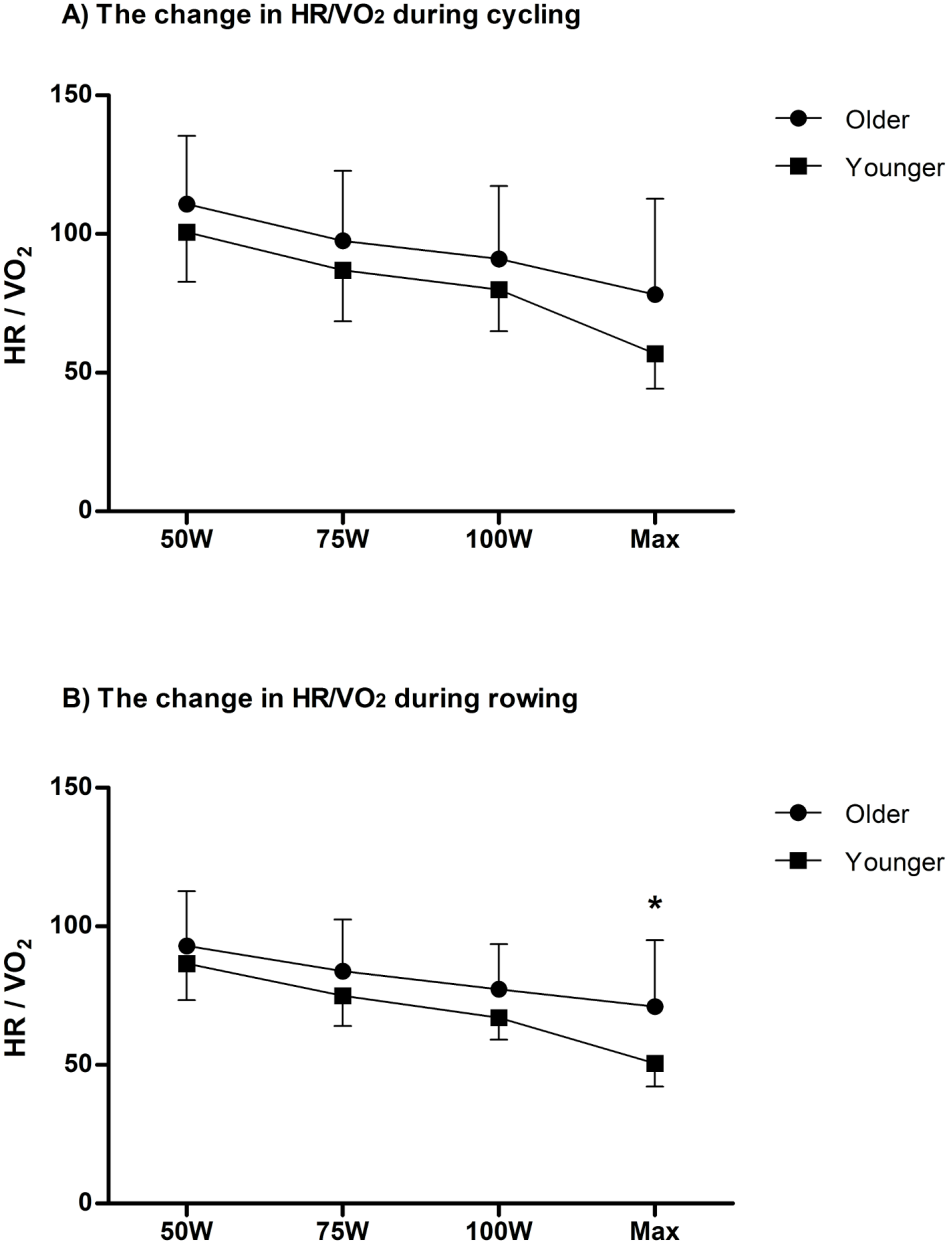
(A) VO_2_ at a given HR. The changes in VO_2/_HR at each workload (50W, 75W, 100W and Max) during cycling (3A) and rowing (3B). * denotes a significant difference between older and younger subjects.

Furthermore, older subjects demonstrated higher HR/VO_2_ ratio, which may indicate a higher HR at a given VO_2_ than younger subjects during rowing (p<0.05). However, there was no significant difference in HR/VO_2_ ratio between groups observed during cycling (p>0.05). Fig 3A and 3B illustrates HR/VO_2_ at a given workload during cycling (3A) and rowing (3B).

## Discussion

Aerobic capacity (VO_2_max) is strongly associated with muscle mass, and rowing VO_2_max was higher than cycling VO_2_max in old and younger athletes. Furthermore, when comparing to older subjects, younger subjects demonstrated a larger change in VO_2_max with rowing compared to cycling. After correcting for the respective active muscle mass being recruited for the specific mode of exercise, older subjects showed no significant difference in VO_2_max during cycling from younger subjects where less muscle mass is being recruited, however, VO_2_max during rowing remained lower in older subjects compared to younger subjects where more muscle mass was being recruited. These findings suggest that VO_2_max is influenced by the total active muscle mass being recruited during exercise, where the more muscle mass involved during exercise, the higher the VO_2_max. However, these data also suggest that VO_2_max in older subjects may be less influenced by muscle mass than younger subjects. In addition, older subjects required higher cardiac effort (higher HR) at a given VO_2_.

Aerobic capacity generally declines with the aging process. In the present experiment, we observed that older subjects demonstrated lower VO_2_max than younger subjects regardless of exercise modes. Generally, VO_2_max declines about 10% per decade [1, 2, 3]. The rate of decline in VO_2_max appears to be greater in athletic or active population than sedentary population [16, 17, 18]. This may result from a higher initial VO_2_max [17]. However, this hypotheses is still not fully tested as results can be inconsistent due to study design (i.e., cross-sectional and longitudinal) and different non-linearity of changes in VO_2_max in athletic/active and sedentary subjects [3, 19].

Over the years, a number of studies have investigated the mechanisms of age-related decline in aerobic capacity. As described by the Fick equation, VO_2_max is determined by maximal cardiac output and arterio-venous O_2_ difference. Age-related declines in VO_2_max are the consequence of decreases in maximal heart rate and stroke volume [20, 21, 22] and maximal arterio-venous O_2_ difference [23]. However, the mechanism for this is not simply explained since there are several confounding factors: diffusion capacity of the lungs, vascular conductance, skeletal muscle mitochondria density and enzyme activity, skeletal muscle mass and training volume.

Skeletal muscle mass loss reaches 10% by age 50 yrs. and decreases further to 30% by age 80 yrs [24, 25]. The natural age-related decline in muscle mass (sarcopenia) and the additional changes in body composition that tend to result from a reduction in physical activity in older subjects both further provoke the age-related declines in VO_2_max [26]. Earlier work by Flegg and Lakatta (1988) assessed 24 hour urinary creatinine excretion to measure muscle mass and reported that skeletal muscle mass was an important determinant for aerobic capacity [12]. In addition, Proctor and Joyner (1997) utilized DEXA scan to assess upper and lower appendicular muscle mass (logical active muscle mass during exercise on treadmill) and found a significant relationship between active muscle mass and VO_2_max [13]. Alternatively, to be more specific to the active muscles involved during exercise in our experiment, we compared the relationship between VO_2_max and LBM for rowing exercise and LMM for cycling exercise. Consistent with previous studies, we observed a strong relationship between muscle mass and VO_2_max in both groups. Further as demonstrated in this study, Secher et al. (1974) observed that subjects showed greater oxygen uptake during combined arm and leg exercise relative to arm or leg exercise alone [27]. Moreover, VO_2_max improved after strength training [26, 28]. Consequently, skeletal muscle mass and VO_2_max are closely related and this phenomenon contributes to the age-related decline in VO_2_max. Nevertheless, we observed that older subjects demonstrated a smaller change in VO_2_max from cycling to rowing than younger subjects even though a similar proportion of muscle mass was added and during rowing correcting for LBM did not account for all the age-related differences in VO_2_max between old and young individuals. This result may suggest that VO_2_max in older subjects is less influenced by skeletal muscle mass than younger subjects. However, further studies are required to truly test this hypothesis.

Interestingly, we observed that during the cycling test (where LMM was significantly different between young and older subjects), there was no difference in VO_2_ max corrected for LMM; whereas during the rowing test where there was no difference in LBM between age groups, there was a significant difference in VO_2_max corrected for LBM. From these results, it appears that the difference in VO_2_max with aging is likely due to other factors such as an age related decline in max HR and consequently cardiac output rather than specific to muscle mass.

Age-related declines in maximal HR and stroke volume in healthy and trained old cohorts are relatively well documented [20, 21, 29]. In addition, decreased cardiac output and reduced blood supply contribute to the age-related decline in VO_2_max [30, 31]. In the present experiment, older subjects had a significantly higher HR at a given VO_2_ during rowing, where they demonstrated a lower VO_2_max, than younger subjects. However, this relationship was not significantly different between groups during cycling as no significant difference in VO_2_max was observed between groups. Therefore, HR seems to be a limiting factor for VO_2_ for older subjects as they may be unable to increase HR to the same extent as such limits the peak VO_2_ they can attain. This hypothesis is consistent with previous finding by Hagberg et al. (1985) reporting that the reduced maximal HR is mainly account for hemodynamic difference between older athletes and younger runners [21].

Although we did not intend to determine the sex differences in this study, subsequent analysis revealed no difference in VO_2_max (L/min) between male and female subjects during cycling and rowing when the data was corrected for LMM and LBM, respectively (p>0.05). Furthermore, the changes in VO_2_max (L/min) from cycling to rowing were not significantly different between groups (p>0.05). These results are in accordance with previous findings by Proctor and Joyner (1997) who reported similar aerobic capacity per muscle mass between highly trained male and female subjects [13].

The present study applied a unique protocol to assess age related differences in VO_2_max, and its’ relationship with active muscle mass. The study provides a cross sectional perspective of the specific population with a practical approach. Nevertheless, a longitudinal study design would provide additional information beyond that which can be extrapolated from cross sectional studies. In this study, rowing VO_2_max was corrected by LBM and cycling VO_2_max by LMM; direct measurement of active muscle mass being recruited was not assessed but rather we theoretically took the approach that rowing exercise would utilize nearly all the lean muscle mass, whereas cycling would recruit primarily leg muscles only. Although a feasible assumption, other muscle groups (e.g., respiratory muscles) will also be recruited during cycling, and this muscle mass was not included in the cycling correction. However, after subsequent analysis which corrected cycling VO_2_max with LBM, there was still no significant difference in VO_2_max between older and younger subjects. Thus, we presumed that this limitation did not influence the outcome. However, exercise-induced adaptations in rowers might influence muscle recruitment (e.g., force production related to concentric versus eccentric contractions) during exercise, and this was not assessed in this study. Although body posture or position might be expected to influence the study outcomes, the impact of posture in this study should be minimal because body posture was similar between exercise modes.

One other potential limitation is the lack of representation of subjects with less muscle mass in the younger group. This may have influenced the relationship between muscle mass and VO_2_max in the younger group.

## Conclusion

Active muscle mass involved during exercise is highly associated with VO_2_max and this relationship may explain partially age-related decline in VO_2_max. However, the influence of muscle mass on aerobic capacity in elderly athletes may be less, compared to younger athletes. Furthermore, central factors, such as the loss of HR and maximal cardiac output with aging clearly contribute to the reductions in aerobic capacity.

## References

[1] Robinson S. Experimental studies of physical fitness in relation to age. Arbeitsphysiologie. 1938; 10: 251–323.

[2] Astrand I. Aerobic work capacity in men and women with special reference to age. Acta Physiol Scand Suppl. 1960; 49: 1–92. 13794892

[3] Hawkins S, Wiswell R. Rate and mechanism of maximal oxygen consumption decline with aging: implications for exercise training. Sports Med. 2003; 33: 877–888. 12974656 10.2165/00007256-200333120-00002

[4] Fitzgerald MD, Tanaka H, Tran ZV, Seals DR. Age-related declines in maximal aerobic capacity in regularly exercising vs. sedentary women: a meta analysis. J Appl Physiol. 1997; 83: 160–165. 9216959 10.1152/jappl.1997.83.1.160

[5] Brown SJ, Ryan HJ, Brown JA. Age-associated changes in VO2 and power output-A cross-sectional study of endurance trained New Zealand cyclists. J Sports Sci Med. 2007; 6: 477–483. 24149481 PMC3794488

[6] Wilson TM, Tanaka H. Meta-analysis of the age-associated decline in maximal aerobic capacity in men: relation to training status. Am J Physiol Heart Circ Physiol. 2000; 278: H829–H834. 10710351 10.1152/ajpheart.2000.278.3.H829

[7] Lakatta EG, Levy D. Arterial and cardiac aging: major shareholders in cardiovascular disease enterprises: Part I: aging arteries: a "set up" for vascular disease. Circulation. 2003; 107: 139–146. 12515756 10.1161/01.cir.0000048892.83521.58

[8] Lakatta EG, Levy D. Arterial and cardiac aging: major shareholders in cardiovascular disease enterprises: Part II: the aging heart in health: links to heart disease. Circulation. 2003; 107: 346–354. 12538439 10.1161/01.cir.0000048893.62841.f7

[9] Tanaka H, Seals DR. Endurance exercise performance in Masters athletes: age-associated changes and underlying physiological mechanisms. J Physiol. 2008; 586: 55–63. 17717011 10.1113/jphysiol.2007.141879 PMC2375571

[10] North BJ, Sinclair DA. The intersection between aging and cardiovascular disease. Circ Res. 2012; 110: 1097–1108. doi: 10.1161/CIRCRESAHA.111.246876 22499900 10.1161/CIRCRESAHA.111.246876 PMC3366686

[11] Mazzeo RS. Exercise and the older adult. ACSM Current Comment [Internet] 2014 [cited 2015 May 9]. Available from: https://www.acsm.org/docs/current-comments/exerciseandtheolderadult.pdf.

[12] Fleg JL, Lakatta EG. Role of Muscle Loss in the Age-Associated Reduction in VO2max. J Appl Physiol. 1988; 65: 1147–1151. 3182484 10.1152/jappl.1988.65.3.1147

[13] Proctor DN, Joyner MJ. Skeletal muscle mass and the reduction of VO2max in trained older subjects. J Appl Physiol. 1997; 82: 1411–1415. 9134886 10.1152/jappl.1997.82.5.1411

[14] Cosgrove MJ, Wilson J, Watt D, Grant SF. The relationship between selected physiological variables of rowers and rowing performance as determined by a 2000 m ergometer test. J Sport Sci. 1999; 17: 845–852. 10.1080/026404199365407 10585164

[15] Rogers MA, Hagberg JM, Martin WH 3rd, Ehsani AA, Holloszy JO. Decline in Vo2max with Aging in Master Athletes and Sedentary Men. J Appl Physiol. 1990; 68: 2195–2199. 2361923 10.1152/jappl.1990.68.5.2195

[16] Eskurza I, Donato AJ, Moreau KL, Seals DR, Tanaka H. Changes in maximal aerobic capacity with age in endurance-trained women: 7-yr follow-up. J Appl Physiol. 2002; 92: 2303–2308. 12015340 10.1152/japplphysiol.01124.2001

[17] Pimentel AE, Gentile CL, Tanaka H, Seals DR, Gates PE. Greater rate of decline in maximal aerobic capacity with age in endurance-trained than in sedentary men. J Appl Physiol. 2003; 94: 2406–2413. 12533496 10.1152/japplphysiol.00774.2002

[18] Ladyga M, Faff J, Burkhard-Jagodzinska K. Age-Related Decrease of the Indices of Aerobic Capacity in the Former Elite Rowers and Kayakers. Biol Sport. 2008; 25: 245–261.

[19] Betik AC, Hepple RT. Determinants of VO2 max decline with aging: an integrated perspective. Appl Physiol Nutr Metab. 2008; 33: 130–140. doi: 10.1139/H07-174 18347663 10.1139/H07-174

[20] Heath GW, Hagberg JM, Ehsani AA, Holloszy JO. A physiological comparison of young and older endurance athletes. J Appl Physiol Respir Environ Exerc Physiol. 1981; 51: 634–640. 7327965 10.1152/jappl.1981.51.3.634

[21] Hagberg JM, Allen WK, Seals DR, Hurley BF, Ehsani AA, Holoszy JO. A hemodynamic comparison of young and older endurance athletes during exercise. J Appl Physiol. 1985; 58: 2041–2046. 4008419 10.1152/jappl.1985.58.6.2041

[22] Fuchi T, Iwaoka K, Higuchi M, Kobayashi S. Cardiovascular changes associated with decreased aerobic capacity and aging in long-distance runners. Eur J Appl Physiol Occup Physiol. 1989; 58: 884–889. 2504587 10.1007/BF02332223

[23] Seals DR, Hagberg JM, Hurley BF, Ehsani AA, Holloszy JO. Endurance Training in Older Men and Women .1. Cardiovascular-Responses to Exercise. J Appl Physiol. 1984; 57: 1024–1029. 6501023 10.1152/jappl.1984.57.4.1024

[24] Lexell J, Taylor CC, Sjostrom M. What is the cause of the ageing atrophy? Total number, size and proportion of different fiber types studied in whole vastus lateralis muscle from 15- to 83-year-old men. J Neurol Sci. 1988; 84: 275–294. 3379447 10.1016/0022-510x(88)90132-3

[25] Deschenes MR. Effects of aging on muscle fibre type and size. Sports Med. 2004; 34: 809–824. 15462613 10.2165/00007256-200434120-00002

[26] Evans WJ, Campbell WW. Sarcopenia and age-related changes in body composition and functional capacity. The Journal of nutrition. 1993; 123: 465–468. 8429405 10.1093/jn/123.suppl_2.465

[27] Secher NH, Ruberg-Larsen N, Binkhorst RA, Bonde-Petersen F. Maximal oxygen uptake during arm cranking and combined arm plus leg exercise. J Appl Physiol. 1974; 36: 515–518. 4826311 10.1152/jappl.1974.36.5.515

[28] Frontera WR, Meredith CN, O'Reilly KP, Evans WJ. Strength training and determinants of VO2max in older men. J Appl Physiol. 1990; 68: 329–333. 2312474 10.1152/jappl.1990.68.1.329

[29] McElvaney GN, Blackie SP, Morrison NJ, Fairbarn MS, Wilcox PG, Pardy RL. Cardiac output at rest and in exercise in elderly subjects. Med Sci Sport Exer. 1989; 21: 293–298. 2733578

[30] Rivera AM, Pels AE 3rd, Sady SP, Sady MA, Cullinane EM, Thompson PD. Physiological factors associated with the lower maximal oxygen consumption of master runners. J Appl Physiol. 1989; 66: 949–954. 2708223 10.1152/jappl.1989.66.2.949

[31] Ogawa T, Spina RJ, Martin WH 3rd, Kohrt WM, Schechtman KB, Holloszy JO et al Effects of aging, sex, and physical training on cardiovascular responses to exercise. Circulation. 1992; 86: 494–503. 1638717 10.1161/01.cir.86.2.494

